# Effect of Hypovitaminosis D on Lipid Profile in Hypothyroid Patients in Saudi Arabia

**DOI:** 10.1155/2020/6640402

**Published:** 2020-12-23

**Authors:** Awad S. Alsamghan, Safar A. Alsaleem, Mohammed A. S. Alzahrani, Ayyub Patel, Ayaz K. Mallick, Salah A. Sheweita

**Affiliations:** ^1^Department of Family and Community Medicine, King Khalid University, Abha, Saudi Arabia; ^2^Department of Medicine, King Khalid University, Abha, Saudi Arabia; ^3^Department of Clinical Biochemistry, Faculty of Medicine, King Khalid University, Abha, Saudi Arabia; ^4^Department of Biotechnology, Institute of Graduate Studies and Research, Alexandria University, Egypt

## Abstract

**Background:**

Hypothyroidism is believed to be associated with dyslipidemia and is considered a risk factor for the development of atherosclerotic cardiovascular diseases (ASCVD). Vitamin D, due to its steroid hormone action, retains cell function and controls the metabolism of lipids. Therefore, the present study was carried out to show the association of the risk factors of ASCVD and deficiency of thyroid hormones and vitamin D levels since no previous studies have been performed on Saudi patients before. *Methodology*. A retrospective cohort study was carried out on 400 hypothyroid patients. Medical records of those patients were followed up and were classified as normal and hypothyroid patients according to their thyroid-stimulating hormone levels. TSH, vitamin D, and lipid profiles were determined using the ELISA technique.

**Result:**

Total cholesterol, triglyceride, and low-density lipoprotein cholesterol levels were significantly higher in hypothyroid patients than those in the normal group. We have found a significant correlation between TSH levels and the risk factors of ASCVD (total cholesterol, triglycerides, and LDL-C). Moreover, a significant correlation between vitamin D levels and the risk factors of ASCVD (total cholesterol, triglycerides, and LDL-C) has been found. In addition, there is a correlation between deficiency of Vit D and low-TSH levels (95% CI 1.092–4.05) indicating a higher risk for the development of ASCVD among those patients.

**Conclusion:**

Hypothyroid and vitamin D-deficient patients must be screened regularly at an early stage to predict and also to prevent cardiovascular diseases. Moreover, an adequate supply of vitamin D and TH should be given to those patients to prevent cardiovascular diseases at an early stage.

## 1. Introduction

Vitamin D insufficiency is associated with cardiometabolic risk factors such as obesity, insulin resistance, hypertension, dyslipidemia, and type 2 diabetes mellitus (DM) [1–3]. Vitamin D deficiency is a global issue occurring in about 30-50 percent of the population of varying age groups [4, 5]. In United States, Canada, and Australia, the mean serum vitamin D levels ranged between 20 and 30 ng/mL pointing towards vitamin D insufficiency [5]. In Brazil, the prevalence of hypovitaminosis D ranged between 5.7% and 52.9% in men over 18 years of age [4]. However, in spite of being a global concern, the reference values for assessment of vitamin D status are controversial. The Brazilian Society of Endocrinology and Metabolism [4] and the Endocrine Society [6] define adequate vitamin D levels at 30 ng/mL, whereas the Institute of Medicine [7] defines it at 20 ng/mL. Apart from this, occupational practices also affect the vitamin D levels as lower levels were reported in night shift workers [8, 9].

Hypothyroidism is known to affect 4-10% of the population, and its incidence is stated to be as high as 10% [10–12]. It is characterised by low levels of thyroid hormones and elevated levels of thyroid-stimulating hormone (TSH). Reduced circulating thyroid hormones have various effects on the cardiovascular system such as decreased cardiac function due to impaired activation of vascular smooth muscles and reduced supply of endothelial nitric oxide. This causes increased vascular resistance [13]. Triiodothyronine (T3) also stimulates the synthesis of renin substrate, hence influencing the renin-angiotensin-aldosterone pathway which in turn may increase the diastolic blood pressure [10].

Dyslipidemia is a chronic metabolic disorder with a negative impact not only on public health but also on the expenses of health care system [14]. Dyslipidemia is one of the leading causes of morbidity and mortality worldwide due to induction of atherosclerotic cardiovascular disease (ASCVD) [15]. It has been found that ASCVD alone is responsible for over 17 million deaths worldwide making it the most common cause of deaths [16]. Following this worldwide trend, the prevalence of dyslipidemia in Saudi Arabia is increasing since it has been reported that between 20 and 40% of Saudi population are dyslipidemic [17]. Numerous factors, such as sociodemographic, nutritional, and economic development and lifestyle patterns, are known to influence the metabolism of lipids and lipoproteins [18]. Thyroid hormones are believed to affect all major metabolic pathways, including lipid oxidation and metabolism control. They regulate the endothelial functions via thyroid hormone receptor- (THR-) 1 and THR-*β*. Activation of THR-*α*1 increases coronary blood flow, decreases coronary resistance in experimental models, and increases the development of nitric oxide in both the endothelial and vascular smooth muscle cells [18].

Downregulation of low-density lipoprotein cholesterol (LDL-C) receptor and iodination of high-density lipoprotein cholesterol (HDL-C) in subclinical hypothyroidism along with elevated total cholesterol significantly alters lipoprotein metabolism, leading to ASCVDD development [15, 19]. Also, decreased LDL-C receptor and cholesterol-*α*-monooxygenase production results in decreased LDL clearance [20].

Hypothyroidism and deficiency of vitamin D lead to hyperlipidemia, thereby raising the risk of cardiovascular diseases. Therefore, this study was performed to investigate the association of dyslipidemia with hypothyroid and/or hypovitaminosis D in Saudi patients since no previous studies have been shown on such relationships. Furthermore, the aim of this study is to focus on early identification of cardiovascular disease risk factors that could be prevented and/or alleviated by hypothyroidism treatment and/or vitamin D dietary supplementation.

## 2. Material and Methods

### 2.1. Study Design and Sample Size

This retrospective cohort study was planned and carried out in the Aseer region of the Kingdom of Saudi Arabia between March 2020 and April 2020 following the approval of the Institutional Ethical Committee. Six centres operated by thyroid clinics have been selected as the data collection source. The sample size for the study was determined to maintain a reasonable *α* error of 5% and *β* error of 0.80 (study power of 80%) with a confidence interval of 95 percent, which was estimated to be 400 cases. Diagnosis of hypothyroidism was conducted on the basis of clinical symptoms of hypothyroidism and serum thyroid-stimulating hormone (TSH) levels greater than 4.5 mIU/L. Newly diagnosed patients that have been in the clinic for the past year were randomly picked. In order to prevent the bias of the investigator, one resident, unaware of the study goals, was assigned to each centre to collect the necessary data from the patient file. Detailed scrutiny of patients with a history of diabetes mellitus, hypertension, renal disorders, and lipid-lowering medicines and vitamin D supplementation was removed from the study. The case records were followed from the time of diagnosis till the collection of data. Their last serum TSH, vitamin D, and fasting lipid profile were reported. Based on their TSH levels, they were classified into two groups: the euthyroid group with serum TSH between 0.5 and 4.5 mIU/L and the hypothyroid group with serum TSH greater than 4.5 mIU/L.

### 2.2. Grouping of the Participants

Based on the lipid profile of all participants, patients in the euthyroid and hypothyroid classes were further divided into two categories: a low- and a high-risk group. The basis for this classification was the LDL-C concentration as it has been identified as the primary therapeutic target for ASCVD by the National Cholesterol Education System–Adult Treatment Plan (NCEP-ATP) as set out in the guidelines provided by the American Heart Association (AHA) [21]. According to their guidelines, five categories have been defined, i.e., LDL − C < 100 mg/dL as ideal, 101–129 mg/dL as above optimum, 130 to 159 mg/dL as moderately high, 160 to 189 mg/dL as high, and 190 mg/dL as very high. In this study, all participants were divided into two groups for the convenience of statistical analysis. Participants with LDL-C up to 129 mg/dL were classified as low risk as this range was within the ideal range, and those with 130 mg/dL and above were at high risk for ASCVD. Similarly, based on the concentration of vitamin D, the participants were divided into two classes according to the recommendations laid down by the Endocrine Society's clinical guidelines [6]. Participants with vitamin D less than 29 ng/mL were classified as a vitamin D deficiency group and those above 30 ng/mL were classified as an adequate vitamin D group. The vitamin D deficiency category included both vitamin D deficiency (>20 ng/mL) and vitamin D insufficiency (21–29 ng/mL).

### 2.3. Biochemical Analysis

Serum total cholesterol (TC), serum triglyceride (TG), and high-density lipoprotein cholesterol (HDL-C) were determined by an enzymatic approach using commercially available kits and an automated analyser. Low-density lipoprotein cholesterol (LDL-C) was calculated using the Friedewald formula. Total vitamin D (25(OH) vitamin D) and serum TSH levels were determined using an electrochemiluminescence method.

### 2.4. Statistical Analysis

All the data obtained were collected and analysed using Statistical Package for Social Sciences (SPSS) version 25.0 (SPSS/PC; SPSS-25.0, Chicago, USA). Parameters of lipid profile, TSH, and vitamin D concentration were expressed as mean (SD). Significance of mean was studied using independent “*t*” test. Correlation between vitamin D and parameters of lipid profile in hypothyroid patients was determined using Pearson's coefficient. Relative risk with 95% confidence interval was determined in order to study the effect of vitamin D deficiency on lipid profile among hypothyroid patients. A *P* value less than 0.05 was considered significant.

## 3. Results

**Figure 1:**
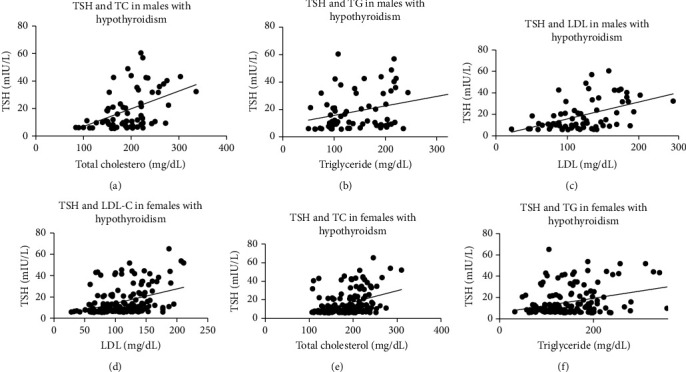
Correlations between thyroid stimulating hormone and lipid profile in hypothyroid male and female patients.

**Figure 2:**
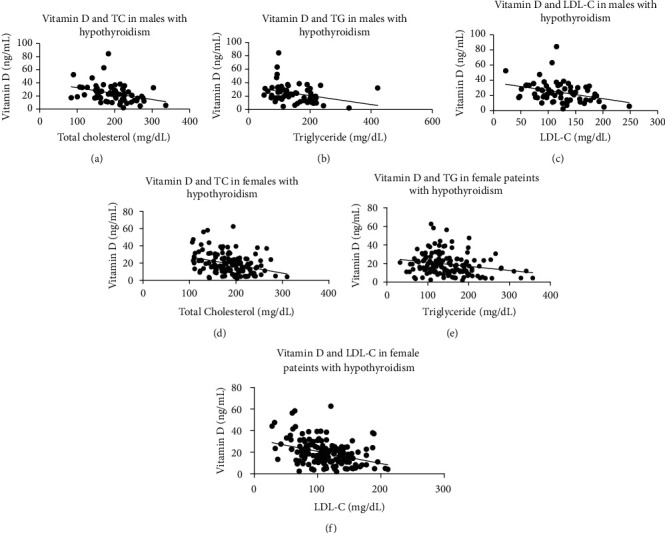
Correlations between vitamin D and lipid profile in hypothyroid male and female patients.

Medical records of 400 patients were followed up, and based on their serum TSH levels, they were divided into two groups, the euthyroid group with 186 patients and the hypothyroid group with 214 patients. As seen in Table 1, the hypothyroid group consisted of 69.2% (148) females and 30.8% (66) males having a combined mean ± SD age of 45.09 ± 15.54 years as compared to 43.78 ± 15.63 years in the euthyroid group. The body mass index (BMI) was significantly higher in hypothyroid patients in comparison to euthyroid patients (*P* < 0.001). No clinical or statistical difference was seen in the systolic and/or diastolic blood pressure of euthyroid and hypothyroid patients (Table 1). The total serum cholesterol, serum triglyceride, LDL-C, and serum TSH levels were elevated in hypothyroid patients whereas the HDL-C was decreased compared to the euthyroid group. All these changes were statistically significant (*P* < 0.05) (Table 1). Hypothyroid male and female had an increased total cholesterol, serum triglyceride, serum LDL, LDL/HDL ratio, and serum TSH levels in comparison to euthyroid females which was statistically significant (*P* < 0.05) (Table 1). On comparing the vitamin D levels in males and females, it was found that females had a lower vitamin D levels in both the euthyroid (*P* < 0.041) and hypothyroid group (*P* < 0.009) whereas in males, it was comparable (Table 1). Comparisons of lipid profile with vitamin D and/or TSH levels in hypothyroid male and female patients were done and are summarized in Figures 1 and 2.

**Figure 3:**
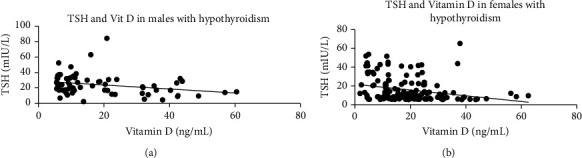
Correlations between TSH and vitamin D in hypothyroid male and female patients.

Gender-based correlation study on TSH and vitamin D with parameters of lipid profile was done in hypothyroid male and female patients (Figures 1 and 2). In order to study the correlation strength, Pearson correlation coefficient (*r*) was determined. Male hypothyroid patients showed a stronger positive correlation between TSH and total cholesterol (*r* = 0.457, *P* < 0.001) (Figure 1(a)), serum triglyceride (*r* = 0.319, *P* = 0.009) (Figure 1(b)) and LDL (0.481, *P* < 0.001) (Figure 1(c)). Hypothyroid females also showed a positive correlation between TSH and total cholesterol (*r* = 0.375, *P* < 0.001) (Figure 1(d)), serum triglyceride (*r* = 0.303, *P* < 0.001) (Figure 1(e)), and LDL (0.378, *P* < 0.001) (Figure 1(f)). In males, the Pearson correlation coefficient between vitamin D with total cholesterol, serum triglyceride, and LDL were -0.336 (*P* = 0.006), -0.320 (*P* = 0.009), -0.344 (*P* = 0.005) and -0.271 (*P* = 0.028) (Figures 2(a), 2(b), and 2(c)) as compared to -0.335 (*P* < 0.001), -0.226 (*P* = 0.006), -0.362 (*P* < 0.001) and -0.265 (*P* = 0.001) (Figures 2(d), 2(e), and 2(f)) respectively in females. A moderate to weak inverse but significant correlation was observed in male patients between vitamin D and increasing TSH (Figure 3(a)) and in females (Figure 3(b)). Interestingly, all these correlations were not found between these parameters in either euthyroid male or female patients (Table 2).

In order to study the risk of ASCVD due to hypovitaminosis D in hypothyroid patients, relative risk was calculated. Based on the concentration of LDL-C on the basis of NCEP-ATP classification, the participants were divided into two groups: the low-risk group for ASCVD with LDL-C up to 129 mg/dL and the high risk group for ASCVD having a serum LDL-C greater than 130 mg/dL. The distribution of hypothyroid and euthyroid patients based on their LDL-C concentration is summarized in Table 3. As depicted in Table 3, 79.4% (170) hypothyroid patients had serum vitamin levels of less than 29 ng/mL and were classified either as either insufficient or deficient as compared to 78% (145) of the euthyroid group. Association of vitamin D deficiency and dyslipidemia in hypothyroid patients was evaluated by determining the relative risk ratio which was found to be 2.103 (95% CI 1.092 to 4.050) indicating that hypothyroid patients with lower vitamin D concentration was more likely to develop dyslipidemia.

## 4. Discussion

Endothelium, the target tissue of thyroid hormones, is adequately responsive to the changes in the level of thyroid hormones [22]. Cardiovascular disease (CVD) is caused by both clinical and subclinical hypothyroidism by disrupting healthy endothelial function by various mechanisms such as inflammation, triggering lipid disorders and oxidative stress [23–25]. In the present study, a strong correlation was observed between increasing TSH and the parameters of lipid profile. The levels of total cholesterol, triglyceride, and LDL-C were higher in the hypothyroid patients which might be due to inhibition of hepatic low-density lipoprotein (LDL) receptors and cholesterol alpha-monooxygenase activity, resulting in decreased clearance of LDL and total cholesterol [26]. Similar dyslipidemia patterns have recently been reported in conjunction with the current research [27, 28]. The decrease in clearance of LDL and total cholesterol in hypothyroid patients might be due to binding of T3 with the sterol regulatory element binding proteins (SREBP), which upregulate the synthesis of LDL receptors along with the regulatory enzymes of cholesterol synthesis, i.e., hydroxymethyl glutaryl CoA (HMG CoA). Therefore, in the absence of T3, the reduction of LDL-C receptors results in an increase in their circulatory levels [29]. In addition, hypothyroidism affects the expression and action of vasorelaxant and vasoconstrictor molecules which control the production of nitric oxide (NO) [24, 30, 31].

The mechanism of increase in the triglyceride level in hypothyroid patients might be attributed to the inhibition of lipoprotein lipase (LPL) activity, a triglyceride hydrolysing enzyme present in the capillary walls of adipose tissue [32–34]. Apart from LPL, a low level of thyroid hormones was found to decrease the activity of hepatic lipase (HL) and cholesterol ester transfer proteins (CETP) which play a crucial role in the reverse cholesterol pathway involving the antiatherogenic HDL-C [32, 33].

Various studies have shown a correlation between hypothyroidism and higher prevalence of obesity [35, 36]. Major weight gain has been associated with even a mild to moderate decrease in thyroid function. In regulating basal metabolism, thyroid hormones play a crucial role, thereby controlling the metabolism of glucose and lipids, the two key fuels for the body. BMI is categorized as healthy weight (BMI 18.5 to 24.9), overweight (BMI 25.0 to 29.9), and obese (BMI 30.0 and above) [37, 38]. In hypothyroid patients, various studies have reported contradictory results about BMI [39]. In this study, the hypothyroid patients had a significantly higher (*P* < 0.005) BMI in comparison to euthyroid patients. Although the exact mechanism by which hypothyroidism causes weight gain is still a matter of investigation, several hypotheses such as increased activity of deiodinase, role of leptin, inflammatory mediators from adipocytes have been postulated [40]. Therefore, obesity resulting from hypothyroidism itself may be a risk factor for development of CVD.

In the current study, vitamin D levels have been found to be lower and inversely correlated with the lipid profile, as vitamin D levels in both euthyroid and hypothyroid patients were below the normal range. Our results are in line with other published studies [41]. The mechanisms of the detrimental effect of reduced vitamin D levels on the lipid profile have been shown by numerous studies in particular on LDL-C and triglycerides [41]. Some of the mechanisms by which vitamin D provides cardioprotective action could be due to foam cell growth prevention, which could lead to rapid relaxation of cardiac myocytes after calcium efflux and nitric oxide release [42]. In support of our results, low vitamin D levels are associated with an increased risk of cardiovascular disease (CVD) and mortality in people with elevated lipid levels [43]. It was also observed that vitamin D inhibited the intracellular NF-*κ*B pathway and in vitro renin synthesis and attenuated the progression of coronary artery disease [44–48]. This inhibition has been shown to reduce cardiovascular disease growth [49–51]. In addition, Al-Rasheed et al. showed that cholecalciferol administration significantly attenuated the production of induced cardiac hypertrophy in mice [52]. Vitamin D also controls cytokine levels, including interleukins (IL-6, IL-8, IL-17A, IL-10) and TGF, and inhibits the pathway of prostaglandins by reducing their receptors and reducing the expression of COX-2 [53]. The cardioprotective function of vitamin D, however, remains controversial, considering all these proposed mechanisms [54]. An inverse relationship between vitamin D and parathyroid hormone (PTH) indicates that hypovitaminosis D may have an indirect effect on CVD. PTH could play a very relevant role in the production of CVD because PTH receptors are abundantly present in cardiac cells and vessels [54].

While conflicting conclusions have been recorded in several observational studies, there appears to be a correlation between vitamin D deficiencies and hypothyroidism. The fact that hypovitaminosis D results in the formation of cytokines and antibodies against thyroid receptors has explained this conflict [55]. It was found in this study that both euthyroid and hypothyroid patients had vitamin D levels below the normal range. The goal of this study was to investigate the increased risk of ASCVD in hypothyroid patients with hypovitaminosis D because both hypothyroidism and vitamin D deficiency are risk factors for hyperlipidemia development. In this study, it was observed that decreases in vitamin D were correlated with increases in total cholesterol, serum triglycerides, and LDL-C in hypothyroid patients, thus signifying their role in CVD development. Although the intensity of the association was mild and statistically relevant, these results are important since hypothyroid patients are already at risk for CVD. Since LDL-C is the primary objective of the National Cholesterol Education Program Adult Treatment Plan (NCEP ATP) for the treatment of dyslipidemia, participants were classified as a high-risk group (LDL − C > 130 mg/dL) and those below 130 mg/dL were classified as a low-risk group. As shown in the present study, the relative risk of ASCVD for the hypothyroid community with hypovitaminosis D (2.103, 95% CI 1.092-4.050) was greater than that of the euthyroid group and significantly increased the risk of ASCVD among hypothyroid patients.

## 5. Conclusion

Hypothyroidism and hypovitaminosis D are known as independent risk factors for the development of cardiovascular diseases. Hypothyroidism and hypovitaminosis D are associated and correlated with total cholesterol, triglycerides, and low-density lipoprotein in Saudi patients. It is also recommended that vitamin D deficiency be checked in hypothyroid patients and that appropriate supplementation may be given if needed. Furthermore, for early detection and/or prediction of cardiovascular disease, screening of thyroid hormone and vitamin D levels should be undertaken at an early stage.

## Figures and Tables

**Table 1:** Changes in lipid profile, vitamin D, and thyroid stimulating hormone in hypothyroid male and female patients.

	Male			Female		
	Euthyroid (*n* = 61)	Hypothyroid (*n* = 66)	Significance	Euthyroid (*n* = 125)	Hypothyroid (*n* = 148)	Significances
Age (years)	46.9 ± 17.35	46.5 ± 15.60	NS (0.90)	42.27 ± 14.56	44.45 ± 15.36	NS (0.233)
BMI	26.06 ± 2.41	29.37 ± 1.71	<0.001	27.07 ± 2.06	29.71 ± 1.94	<0.001
Systolic BP (mmHg)	125.88 ± 14.58	127.60 ± 14.26	NS (0.529)	122.21 ± 14.06	121.88 ± 16.43	NS (0.87)
Diastolic BP (mmHg)	73.88 ± 9.87	76.42 ± 9.87	NS (0.18)	73.09 ± 9.33	71.05 ± 9.65	NS (0.09)
Total cholesterol (mg/dL)	174.16 ± 40.40	193.94 ± 50.86	0.017	169.56 ± 32.20	185.34 ± 40.74	<0.001
Serum triglyceride (mg/dL)	131.80 ± 47.07	149.71 ± 66.17	*NS* (0.08)	122.29 ± 48.84	147.05 ± 58.93	<0.001
HDL-C (mg/dL)	43.97 ± 8.35	42.26 ± 8.82	0.265	45.14 ± 8.07	43.28 ± 9.49	NS (0.09)
LDL-C (mg/dL)	103.87 ± 34.91	121.74 ± 43.62	0.012	100.02 ± 29.58	112.67 ± 36.93	0.002
LDL/HDL ratio	2.42 ± 0.85	3.00 ± 1.23	0.003	2.286 ± .76	2.75 ± 1.13	<0.001
Vitamin D (ng/mL)	25.17 ± 15.73	24.00 ± 13.64	0.654	20.89 ± 11.96	19.26 ± 11.51	NS (0.65)
TSH mIU/L	1.98 ± 0.92	19.07 ± 14.32	<0.001	2.22 ± 1.01	15.79 ± 13.18	<0.001

**Table 2:** Correlations between lipid profile and vitamin D and thyroid stimulating hormone in euthyroid male and female patients.

	Male		Female	
	Pearson co-efficient (r)	*P* value	Pearson coefficient (*r*)	*P* value
TSH and total cholesterol	0.18	NS (0.16)	-0.05	NS (0.52)
TSH and serum triglyceride	-0.99	NS (0.19)	0.03	NS (0.73)
TSH and LDL-C	0.93	NS (0.13)	-0.01	NS (0.88)
TSH and vitamin D	-0.17	NS (0.89)	-0.11	NS (0.22)
Vitamin D and total cholesterol	-0.05	NS (0.68)	-0.04	NS (0.68)
Vitamin D and serum triglyceride	-0.24	NS (0.06)	-0.02	NS (0.812)
Vitamin D and LDL-C	-0.08	NS (0.55)	-0.02	NS (0.86)

**Table 3:** A cross-tabulation showing number of participants and relative risk with a 95% confidence interval between vitamin D deficiency and development of high-risk dyslipidemia among the euthyroid and hypothyroid groups.

Vitamin D levels	Dyslipidemia		Relative risk	95% confidence interval
	High risk	Low risk		
Euthyroid group (*n* = 186)				
Deficiency (*n* = 145)	22	123	0.622	0.32–1.20
Sufficient (*n* = 41)	10	31		
Hypothyroid group (*n* = 214)				
Deficiency (*n* = 170)	65	105	2.10	1.09–4.05
Sufficient (*n* = 44)	8	36		

## Data Availability

All data are available and included in the text.
